# Surveying physical therapists' understanding of benign paroxysmal positional vertigo

**DOI:** 10.3389/fresc.2023.1228453

**Published:** 2023-08-17

**Authors:** Regan G. Harrell, Rebecca Hart, Joanna C. Jen, Susan L. Whitney

**Affiliations:** ^1^ Department of Physical Therapy, The University of Pittsburgh, Pittsburgh, PA, United States; ^2^ Department of Neurology, Icahn School of Medicine at Mount Sinai, New York, NY, United States

**Keywords:** BPPV, subjective BPPV, vestibular agnosia, vestibular rehabilitation, physical therapy (Canalith-repositioning maneuver)

## Abstract

**Introduction:**

Benign paroxysmal positional vertigo (BPPV) is a common condition with disabling symptoms that is diagnosed and effectively treated at the bedside. Our encounter with patients experiencing prolonged BPPV who may not have received appropriate physical therapy prompted us to explore barriers to the diagnosis and treatment for BPPV among physical therapists, which has not been extensively investigated. We hypothesize that a potential barrier may be a lack of understanding of subtle symptoms of BPPV that deviate from the classical presentation. The gold standard for diagnosing definite BPPV is subjective dizziness or vertigo with nystagmus in response to positional testing. There are variants of BPPV including subjective BPPV (subjective dizziness or vertigo without nystagmus) and vestibular agnosia (nystagmus without subjective dizziness or vertigo) that do not meet the diagnostic criteria for definite BPPV but are equally responsive to the same repositioning maneuvers. The purpose of this project was to survey physical therapists for their understanding of BPPV including subjective BPPV and vestibular agnosia.

**Methods:**

A panel of experts created a 16-question survey, designed for physical therapists, with three categories: (1), inquiring if they treat persons with BPPV, (2) three clinical vignettes for definite BPPV, subjective BPPV, and BPPV with vestibular agnosia, and (3) demographic information. Data collection occurred at two large physical therapy meetings, one of which was a national professional meeting and the other was a professional continuing medical education course geared towards advancing vestibular rehabilitation skills.

**Results:**

There were 426 people who completed the survey, 364 of whom treat BPPV in their practice. In the first clinical vignette created to assess the respondents' understanding of definite BPPV, 229 (62%) of respondents would always assess a patient for BPPV based on complaints of a “room spinning” vertigo from head movement. When asked if the complaint was lingering “lightheadedness or feelings of imbalance” from head movement, only 158 (43%) reported they would perform positional testing to reassess. In the BPPV variant vignettes, 187 (51%) identified the patient with subjective BPPV as having BPPV and 305 (85%) identified the patient with vestibular agnosia as having BPPV.

**Discussion:**

The results of this survey demonstrate gaps in knowledge regarding BPPV across practice settings and experience, with opportunities to bridge these gaps to improve treatment for BPPV.

## Introduction

Benign Paroxysmal Positional Vertigo (BPPV) has a lifetime prevalence of 2.4% and is the most common diagnosis for recurrent dizziness or vertigo (1). BPPV is a mechanical inner ear disorder caused by displaced otoconia in the semicircular canal(s) (2). Classic symptoms reported with BPPV include vertigo—a room-spinning sensation of dizziness- with a change in head position often with associated gait instability and nausea (3). Transient subjective dizziness or vertigo with nystagmus in the plane of the involved semicircular canals from positional testing are diagnostic of definite BPPV (2). If left untreated, BPPV is correlated with a decrease in activities of daily living scores, an increased rate of falling, and increased rates of depression (3–6). According to the American Academy of Otolaryngology-Head and Neck Surgery (AAHN) 2018 Clinical Practice Guidelines and recommendations by the American Academy of Neurology, positional tests are the gold standard for diagnosing BPPV, with strong recommendations for treating BPPV with canalith repositioning maneuvers (3, 7).

The criteria for definite BPPV include transient subjective positional dizziness or vertigo and positional nystagmus corresponding to the plane of the semicircular canal that is tested. There are two alternate variants of BPPV that do not meet the criteria for definite BPPV: subjective BPPV and BPPV with vestibular agnosia (8, 9). Patients with subjective BPPV report dizziness or vertigo in response to positional testing but do not have the corresponding positional nystagmus (8, 10–17). Patients with vestibular agnosia have the correct nystagmus pattern in the positional test but do not report any symptoms of dizziness (9, 18, 19).

Although other healthcare providers including neurologists, otolaryngologists, primary care physicians, and audiologists receive training in the diagnosis and management of BPPV, patients with BPPV are routinely referred for physical therapy. Diagnosing and treating BPPV is within the Physical Therapy Guide To Practice, which was compiled by the American Physical Therapy Association as a resource describing physical therapy practice and is used as the guideline for developing physical therapy curriculum (20). Diagnosing and management of BPPV is a skill all physical therapists are exposed to in their entry level education. Yet, we have encountered patients who suffered prolonged symptoms from BPPV despite physical therapy. We hypothesized that one of the potential barriers may be limited understanding of subtle manifestations of BPPV. The aim of this study was to test our hypothesis by assessing the current understanding within the physical therapy community of definite BPPV, subjective BPPV, and BPPV with vestibular agnosia. We also examined if the survey responses correlated with practice settings or clinical experience.

## Methods

A panel of experts developed the survey with Qualtrics (Qualtrics, Provo, UT) to explore the familiarity of physical therapists with BPPV and its variants. The panel included four members in academic tertiary medical centers in metropolitan areas with a large referral base: two physical therapists, a vestibular neurologist, and a research assistant. The University of Pittsburgh Biomedical IRB approved the study (STUDY23020028). All respondents provided consent to complete the survey (Appendix).

There were three sections with a total of 16 questions. The first section asked if the physical therapist treats patients with BPPV and, if they do not, to whom they would refer the patient. The second section had three short case vignettes on definite BPPV, subjective BPPV, and BPPV with vestibular agnosia. The third section included demographic information. Questions in the second section on clinical vignettes probed how likely the therapists were to perform the correct positional testing on a 5-point Likert scale: never, sometimes, about half the time, most of the time, and always. The triggers in the vignettes involved head movement in the vertical plane to implicate the posterior semicircular canals, for which the Dix-Hallpike positional testing should be performed. For the clinical vignettes addressing the subtle presentations the respondents answered the clinical questions with what diagnosis they thought the patient presented with: Functional Dizziness (e.g., Persistent Postural Perceptual Dizziness, Mal de Debarquement Syndrome), vestibular migraine, BPPV, or unable to make a physical therapy diagnosis. If the respondent answered, “I am unable to make a physical therapy diagnosis”, they then answered a series of follow-up questions including “If you are unable to make a physical therapy diagnosis, what would you do?” with answers including recommend Meclizine/Dramamine, perform repositioning exercise as dictated by the involved canal, or refer out to another provider. Any respondent who answered “refer out to another provider” then selected which specialty they would refer the patient to.

The final section of the survey included demographic information, including current practice setting and length of time working as a physical therapist. Respondents completed the survey via QR code at two large physical therapy conferences with physical therapists and physical therapy students from across the United States and from varying practice settings.

Descriptive statistics were completed on the entire sample. Sub-analyses were conducted based on years of practice as a physical therapist and practice area. *T*-tests were calculated to assess differences between physical therapists with ≤11 years of experience versus those with >11 years of experience. Kruskal-Wallis test statistics were calculated to assess for differences in response rate based on clinical practice settings. All statistical analyses were completed with SPSS (Version 27.0), with *α *= 0.05 used for the level of significance.

## Results

There were 426 people who completed the survey. Table 1 demonstrates the areas of practice for those who completed the survey. The average number of years for those who do not treat people with BPPV was 7 years and those who treat people with BPPV was 11 years. For those that treat BPPV in their practice, there were 364 clinicians with a mean of 11 years of practice experience. Respondents who treat BPPV came from clinical practice areas including academics, acute care, home health, inpatient rehabilitation, outpatient neurological/vestibular, outpatient orthopedic, skilled nursing, sports, and students within the survey respondents. In the group of respondents who do not treat BPPV (*n* = 60), see Table 1, there was a similar range of practice settings represented and there was also one respondent who works in an oncology setting.

**Table 1:** Demographics of the physical therapists and physical therapist students who completed the BPPV survey.

Physical therapists and physical therapist students who completed the survey	Frequency (percentage)	
	426	
Practice setting	Percentage of the sample who do not treat patients with BPPV (*n* = 62)	Percentage of the sample who treat persons with BPPV (*n* = 364)
Academic/Research	8 (13%)	10 (3%)
Acute care	5 (9%)	50 (14%)
Home health	6 (10%)	7 (2%)
Inpatient rehabilitation	4 (5%)	39 (10%)
Oncology	1 (1%)	0 (0%)
Outpatient neurologic/vestibular	1 (1%)	152 (42%)
Outpatient orthopedics	3 (5%)	74 (21%)
Pediatrics	0 (0%)	0 (0%)
Sports	0 (0%)	3 (1%)
Skilled nursing facility	1 (1%)	1 (1%)
Women's health	0 (0%)	0 (0%)
In school	33 (53%)	24 (6%)

**Figure 1:**
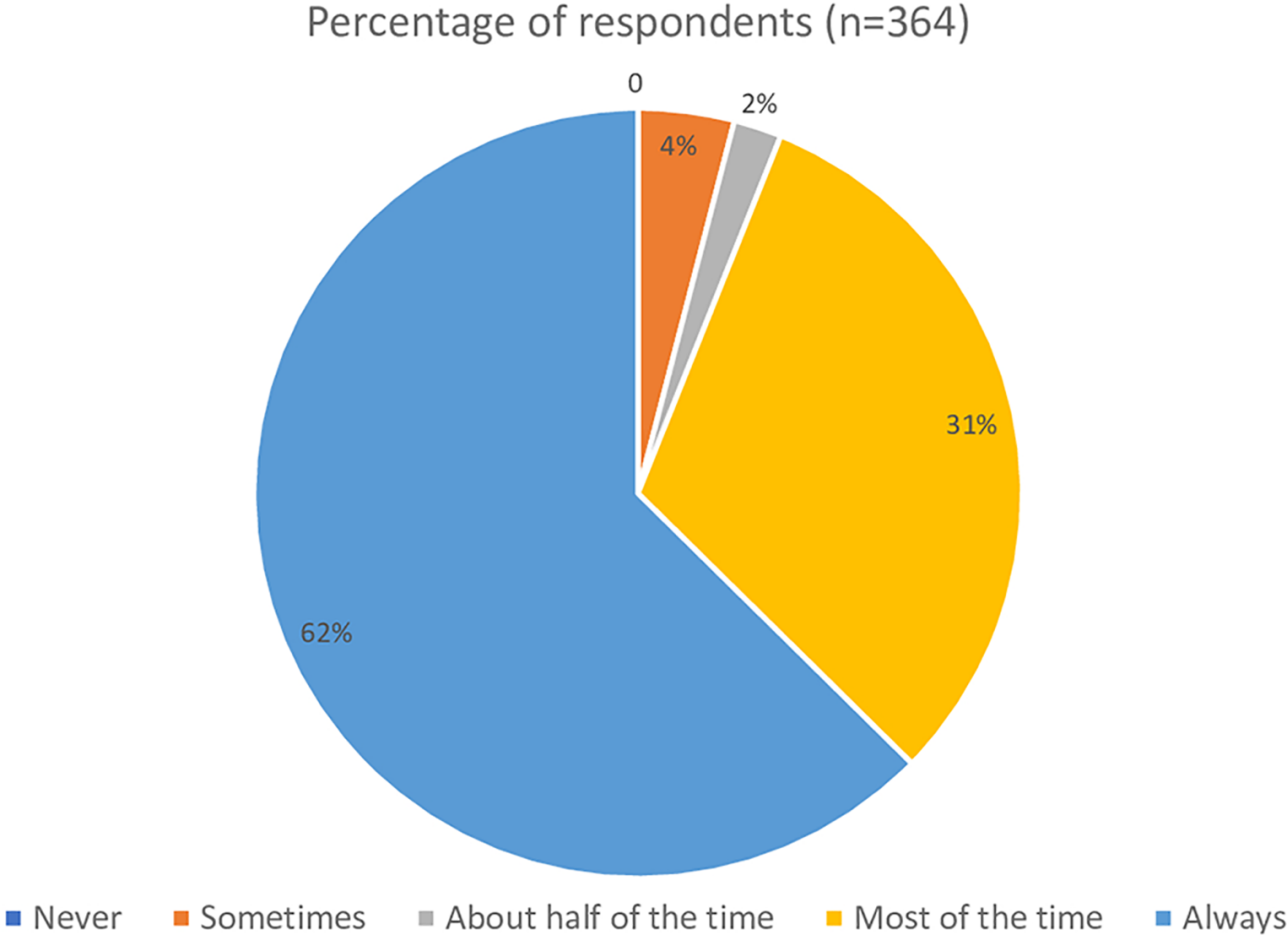
Responses from the first clinical vignette “A 65-year-old male presents to your clinic with complaints of brief spinning dizzy spells from getting in and out of bed, looking up and down, walking, and physical activities in general. Would you assess this patient using positional testing (such as the Dix-Hallpike)?”.

**Figure 2:**
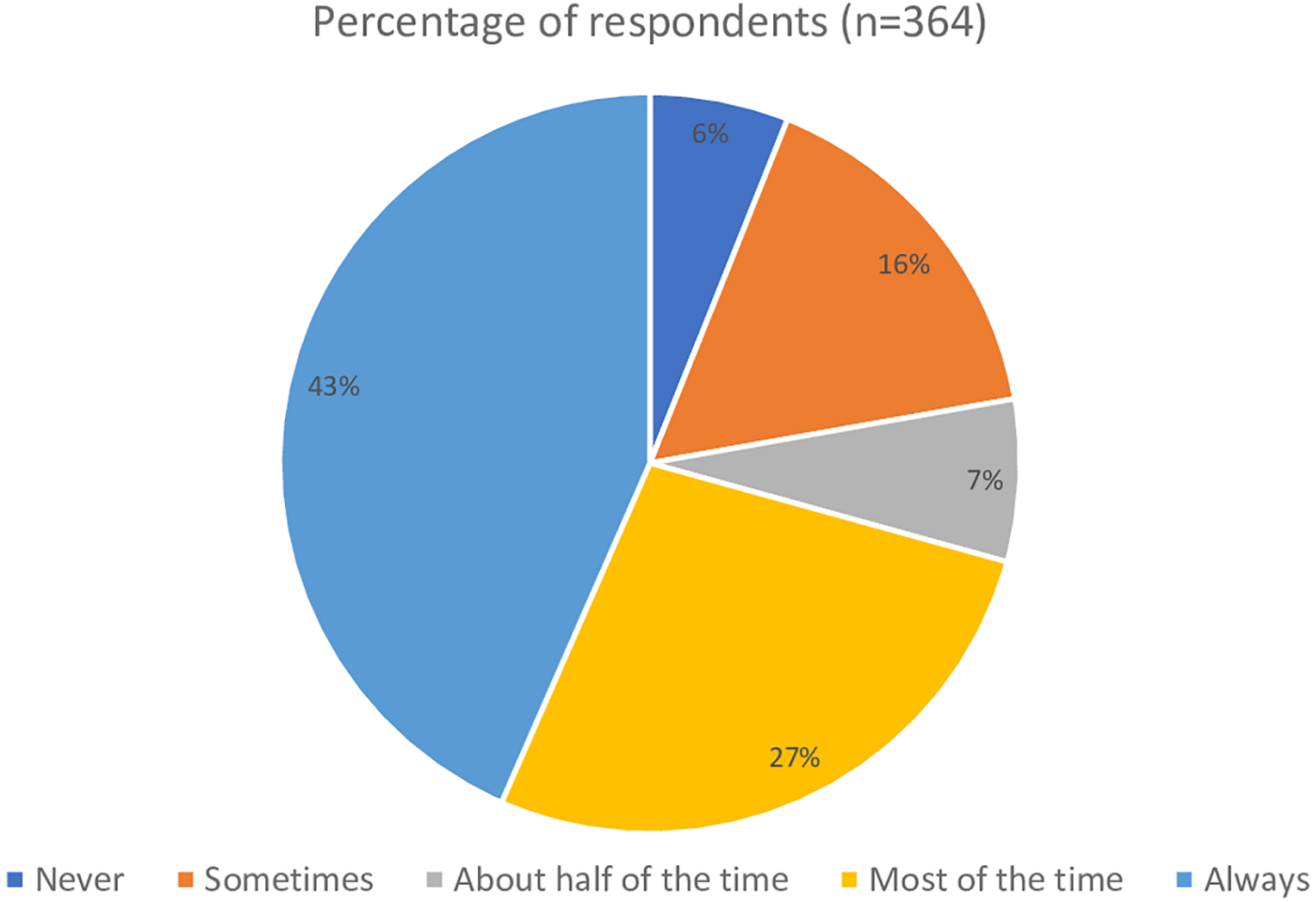
Responses from the follow up question to the first clinical vignette “When the patient returns the next visit following treatment for BPPV, he continues to complain of being off balance with slight non-spinning light-headedness in response to getting in and out of bed, looking up and down, walking, and physical activities in general. Would you reassess this patient using positional testing (such as the Dix-Hallpike)?”.

**Figure 3:**
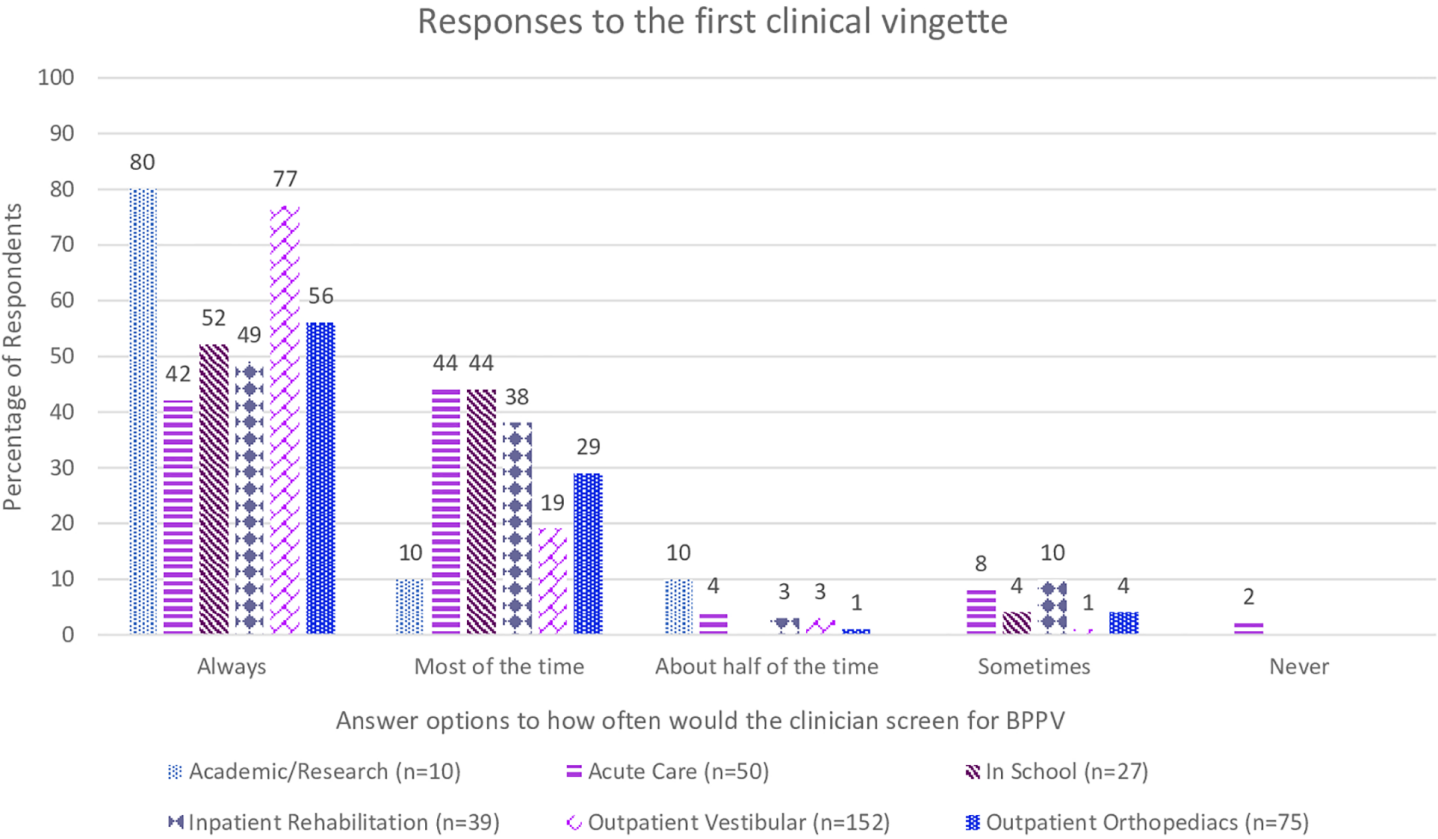
Responses from the first clinical vignette “A 65-year-old male presents to your clinic with complaints of brief spinning dizzy spells from getting in and out of bed, looking up and down, walking, and physical activities in general. Would you assess this patient using positional testing (such as the Dix-Hallpike)?” based on practice area of the respondent.

**Figure 4:**
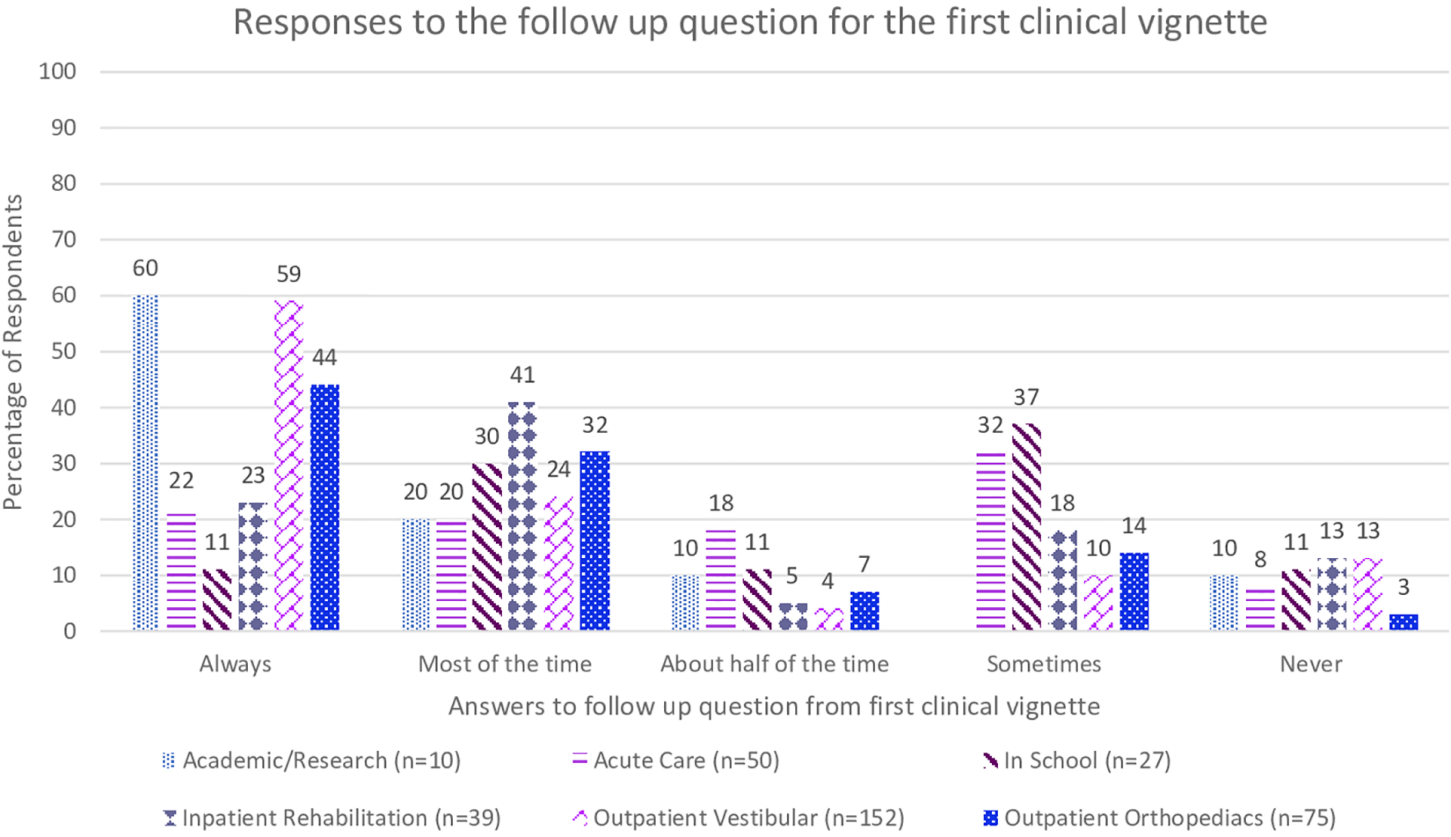
Responses from the follow up question to the first clinical vignette “When the patient returns the next visit following treatment for BPPV, he continues to complain of being off balance with slight non-spinning light-headedness in response to getting in and out of bed, looking up and down, walking, and physical activities in general. Would you reassess this patient using positional testing (such as the Dix-Hallpike)?” based on practice area of the respondent.

The first clinical vignette investigated the respondents' understanding of definite BPPV and their likelihood to perform positional testing to correctly diagnose the condition. Seen in Figure 1, 229 (62%) of respondents would always perform positional testing, with 111 (31%) who would perform positional testing most of the time. The respondents were asked if they would reassess the same patient for BPPV at a return visit if the symptoms reported included “off balance and non-spinning dizziness” in response to the same positional triggers, which would suggest subjective BPPV with residual debris (Figure 2). Only 158 (43%) of the respondents said they would always reassess the above patient for BPPV, and 100 (27%) responded that they would reassess most of the time. While there was a decrease in the number of responses who would always screen this patient for BPPV, it was not significant (*t* = 2.13, *p* = 0.49, *d* = 0.15). When the respondents' answers were analyzed broken down by years of experience, there was not a significant difference in responses between groups (*t* = 1.56, *p* = 0.19). Figure 3 shows the responses to the first clinical vignette based on practice area for those with greater than ten respondents. There was not a significant difference in responses based on clinical practice [H(5) = 5.1, *p* = 0.2]. The trend appeared that those in academic/research (80%) and outpatient vestibular/neurological clinics (77%) had the highest rate of always assessing for BPPV. When reassessing the same patient for BPPV, there was a reduction in the number of respondents always screening for BPPV and an increase across all practice areas of respondents never screening for BPPV (Figure 4).

**Figure 5:**
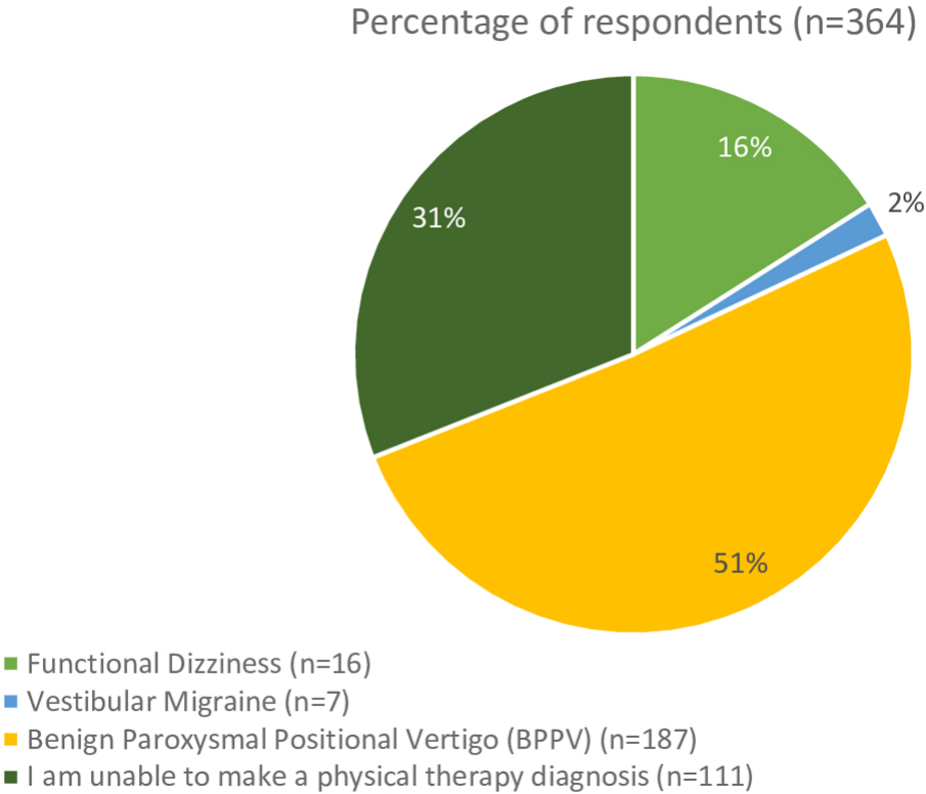
Responses from the clinical question “A patient reports spinning vertigo that lasts 10 s without nystagmus in the Dix-Hallpike position. What is your diagnosis?”.

The second clinical vignette asked the respondents for their diagnosis of a patient with subjective dizziness without nystagmus in response to positional testing. Overall, 187 (51%) respondents selected subjective BPPV, 111 (31%) were unable to make a diagnosis, and the remainder chose vestibular migraine and functional dizziness (Figure 5). In the 111 who could not make a PT diagnosis, 87 would refer this patient to another provider. There was not a significant difference in response rates based on years of experience (*t* = 1.39, *p* = 0.13). Based on practice setting, 30% of those in an academic/research setting, 44% of acute care, 42% of those in home health, 31% of those in school, 61% of those in outpatient neurologic/vestibular, 59% of those in outpatient orthopedic, and 67% of those in sports settings diagnosed the patient with BPPV. There was not a significant difference in response rates based on clinical practice setting [H(5) = 4.36, *p* = 0.36]. There were 40% of those in academic/research, 36% in acute care, 42% of those in home health, 13% in school, 41% of those inpatient rehabilitation, 29% in outpatient neurologic/vestibular, and 23% of those in outpatient orthopedic settings were not able to make a physical therapy diagnosis. The providers they would refer to include Otolaryngology, Neurology, Primary Care, Vestibular Physical Therapy, and Audiology.

**Figure 6:**
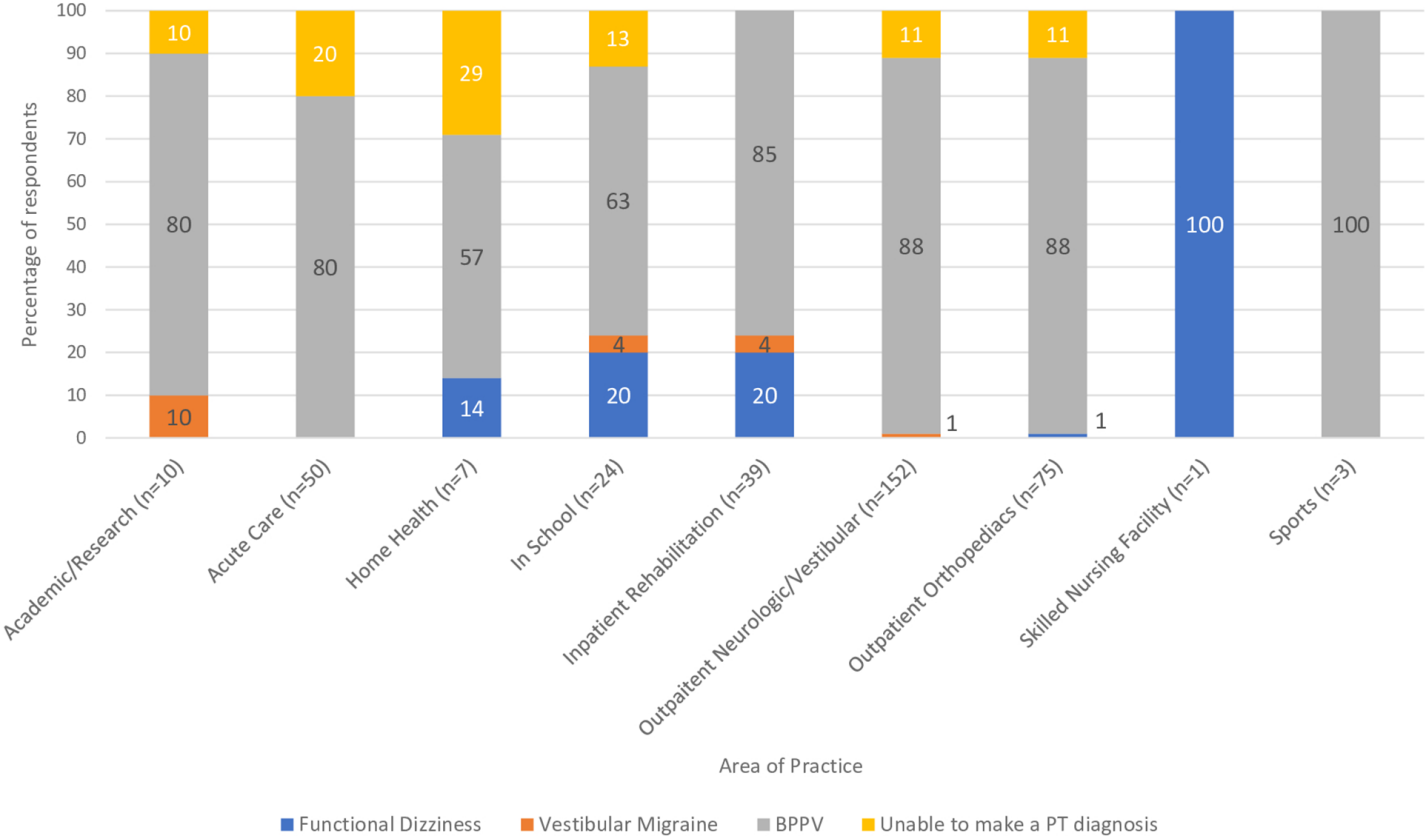
Responses from the clinical question, based on practice setting, “If you observe torsional upbeating nystagmus that fatigues in response to Dix-Hallpike head hanging, but they report no spinning. What is your diagnosis?”.

The third clinical vignette focused on BPPV with vestibular agnosia. 305 (85%) respondents correctly diagnosed this vignette as having BPPV, while 9 (2%) stated it was functional dizziness, 5 (1%) stated it was vestibular migraine, 45 (12%) could not make a PT diagnosis. Figure 6 illustrates the preferred diagnosis of the third vignette by clinical practice setting. There was not a significant difference in responses based on years of experience (*t* = 0.77, *p* = 0.25). There was not a significant difference in response rates based on clinical practice setting [H(5) = 1.23, *p* = .87]. Of the 45 respondents who could not make a PT diagnosis, 42 would refer them to another provider. These providers included Otolaryngology, Neurology, Primary Care, and Vestibular Physical Therapy.

## Discussion

BPPV is the most common cause of recurrent dizziness and vertigo. There is often delay in the diagnosis and treatment for BPPV with documented underutilization of positional testing by physicians in primary care and emergency departments (21). Since dizzy patients are commonly referred for physical therapy, we aim to investigate the level of familiarity with BPPV among physical therapists, which has not been studied previously. We hypothesized that there may be limited familiarity with variants of BPPV with subtle manifestations. We further hypothesized that clinical experience may have a positive correlation with knowledge regarding BPPV.

The importance of a high clinical suspicion for BPPV is that positional testing specifically Dix-Hallpike positioning for posterior canal involvement would dictate treatment. Subjective BPPV and BPPV with vestibular agnosia are two variants that do not fully meet the diagnostic criteria for definite BPPV. In a cohort of 204 patients with BPPV, 64 had subjective BPPV, and there was no significant difference in treatment response between those with classic BPPV and those with subjective BPPV (8). Jung and Kim treated 134 persons with BPPV, 33 of whom had subjective BPPV (22); they found no significant difference in recovery rates between those with or without positional nystagmus (3, 22). Uz et al. found that older adults with subjective BPPV had improved quality of life after the Epley maneuver (17). If recognized and correctly diagnosed, BPPV along with its variants is effectively treated by repositioning maneuvers that physical therapists are trained to perform.

In the first part of the first clinical vignette, which presented a definite BPPV case, although 62% of respondents would always assess the patient for BPPV, there is still 38% that would not always assess a person with such classic presentation for BPPV. The second part of the first vignette is a common presentation of residual BPPV that is incompletely treated, in that the patient no longer has positional nystagmus on exam yet continues to be bothered by the same positional triggers. The correct response should be to have a high clinical suspicion for BPPV and to always perform positional testing for a treatable condition. Yet the survey showed further decrease in the number of respondents who would always assess the patient for BPPV. The change in responses is an almost 20% reduction in those who would always evaluate this patient for BPPV, while not significant this trend is still concerning as these patients will not receive appropriate treatment. Furthermore, if physical therapists could not recognize incompletely treated BPPV, it would be even less likely that they would recognize subjective BPPV, as presented in the second vignette. The AAHN Clinical Practice Guidelines recommend testing for BPPV in those who “report a history of vertigo provoked by changes in head position relative to gravity” (3). While “room spinning” dizziness is considered the hallmark symptom of posterior canal BPPV, others have reported persons with BPPV endorsing light-headedness, dizziness, sinking, floating, nausea, or feeling off balance (1, 23, 24). BPPV is a vestibular abnormality and can result in an increased risk of falling and impairments in activities of daily living (3–5). Adults over 40 with vestibular dysfunction have a 12-fold increase in the odds of falling (25). BPPV has been correlated to falls (4, 6, 19).

The second clinical vignette sought to capture clinicians' understanding of subjective BPPV. From the current study, only 51% of respondents could identify subjective BPPV, and 31% could not make the diagnosis. The lack of nystagmus in positional testing in people with subjective BPPV likely contributes to the low diagnosis rate. It is hypothesized that subjective BPPV represents a subthreshold amount of dislodged debris that is insufficient to drive vestibular nystagmus but enough to cause subjective dizziness (2, 15, 26).

The third vignette focused on BPPV with vestibular agnosia. Vestibular agnosia was first described in 2021 by Calzolari et al. and is defined as “a loss of vertigo sensation in patients with preserved inner ear functioning” (9). This phenomenon has been primarily identified in a traumatic brain injury population but has also been reported in older adults (9, 19, 27). A person with vestibular agnosia would have positional nystagmus in the positional tests but will not report vertigo. In the current survey, the third clinical vignette sought to capture the clinician's understanding of BPPV with vestibular agnosia. Of the respondents, 85% identified the scenario as BPPV, with only 12% unable to make a diagnosis. The percentage of respondents who correctly identified this vignette as BPPV (84%) is higher than those who could identify subjective BPPV (51%). The presence of an objective sign with positional nystagmus corresponding to the stimulated semicircular canal in BPPV with vestibular agnosia likely contributes to the consideration of BPPV. A potential explanation is that subjective BPPV and BPPV with vestibular agnosia are relatively recent designations that have not been disseminated.

Several direct and indirect costs occur to the patient by not correctly identifying subjective BPPV. The US's average cost of diagnosing and treating BPPV is $2 billion annually, secondary to unnecessary imaging and referrals to specialists (3). A systematic review by Kovacs et al. found that in persons with vestibular vertigo, 61.3% of them had more than two specialist consultations before receiving a diagnosis (28). Up to 50% of persons with vertigo received a CT scan, and 18.6% received an MRI (28). The indirect costs of untreated vertigo include 63.3% of persons with vertigo losing working days related to their symptoms and 5.7% leaving the workforce because of their symptoms (29).

According to the US Bureau of Labor Statistics, there are approximately 225,350 physical therapists in the United States (30). The respondents to our survey represent a small sample of the total physical therapists in the country. Due to the continuing education nature of the meetings where the survey occurred, this may skew the sample to therapists more interested in advancing their knowledge and skill set that the true understanding of the variants of BPPV would most likely be lower than reported in this survey. We had hypothesized that those working in outpatient neurological/vestibular clinics with more years of experience would have higher rates of always screening for BPPV and recognizing the variants of BPPV. The results from our survey showed that experience did not correlate with the correct responses.

## Conclusion

There is ongoing effort to improve the recognition and treatment of BPPV by front-line practitioners in primary care and emergency departments (26, 31). Our study only analyzed physical therapists' understanding of definite BPPV and the relatively recently described variants with more subtle presentation to demonstrate that there is a gap in knowledge regarding BPPV. Potential methods of improving recognition of BPPV would be increasing the training for BPPV diagnosis and management in the physical therapy curriculum and increasing access to vestibular specific continuing education courses post licensure across clinical settings and experience. We also propose greater collaboration and communication between the referring physician and physical therapist to improve the care of patients with BPPV and other vestibular disorders.

## Data Availability

The raw data supporting the conclusions of this article will be made available by the authors, without undue reservation.

## References

[1] von Brevern M Radtke A Lezius F Feldmann M Ziese T Lempert T Epidemiology of benign paroxysmal positional vertigo: a population based study. J Neurol Neurosurg Psychiatry. (2007) 78(7):710–5. 10.1136/jnnh2006.100420 17135456 PMC2117684

[2] von Brevern M Bertholon P Brandt T Fife T Imai T Nuti D Benign paroxysmal positional vertigo: diagnostic criteria. J Vestib Res. (2015) 25(3–4):105–17. 10.3233/VES-150553 26756126

[3] Bhattacharyya N Gubbels SP Schwartz SR Edlow JA El-Kashlan H Fife T Clinical practice guideline: benign paroxysmal positional vertigo (update). Otolaryngol Head Neck Surg. (2017) 156(3_suppl):S1–S47. 10.1177/0194599816689667 28248609

[4] Donovan J De Silva L Cox H Palmer G Semciw AI. Vestibular dysfunction in people who fall: a systematic review and meta-analysis of prevalence and associated factors. Clin Rehabil. (2023) 37(9):1229–47. 10.1177/02692155231162423 37036433

[5] Fancello V Hatzopoulos S Santopietro G Fancello G Palma S Skarżyński PH Vertigo in the elderly: a systematic literature review. J Clin Med. (2023) 12(6):2182. 10.3390/jcm12062182 36983184 PMC10058392

[6] Pauwels S Casters L Lemkens N Lemmens W Meijer K Meyns P Gait and falls in benign paroxysmal positional vertigo: a systematic review and meta-analysis. J Neurol Phys Ther. (2023) 47(3):127–38. 10.1097/NPT.0000000000000438. 36897200 PMC10521788

[7] Fife TD Iverson DJ Lempert T Furman JM Baloh RW Tusa RJ Practice parameter: therapies for benign paroxysmal positional vertigo (an evidence-based review): report of the quality standards subcommittee of the American academy of neurology. Neurology. (2008) 70(22):2067–74. 10.1212/01.wnl.0000313378.77444.ac 18505980

[8] Balatsouras DG Korres SG. Subjective benign paroxysmal positional vertigo. Otolaryngol Head Neck Surg. (2012) 146(1):98–103. 10.1177/0194599811425158 21998085

[9] Calzolari E Chepisheva M Smith RM Mahmud M Hellyer PJ Tahtis V Vestibular agnosia in traumatic brain injury and its link to imbalance. Brain. (2021) 144(1):128–43. 10.1093/brain/awaa386 33367536 PMC7880674

[10] Celis-Aguilar EM Medina-Cabrera CA Torrontegui-Zazueta LA Núñez-Millán BX Castro-Bórquez KM Obeso-Pereda A Short-term effect of epley maneuver as treatment for subjective benign paroxysmal positional vertigo. Indian J Otolaryngol Head Neck Surg. (2022) 74(Suppl 1):545–9. 10.1007/s12070-020-02320-y 36032873 PMC9411366

[11] González-Aguado R Domènech-Vadillo E Álvarez-Morujo de Sande MG Guerra-Jiménez G Domínguez-Durán E. Subjective benign paroxysmal positional vertigo in patients with osteoporosis or migraine. Braz J Otorhinolaryngol. (2020) 86(1):83–90. 10.1016/j.bjorl.2018.10.003 30482521 PMC9422745

[12] Harmat K Tamás LT Schubert MC Gerlinger I Komoly S Büki B. Prevalence of and theoretical explanation for type 2 benign paroxysmal positional vertigo. J Neurol Phys Ther. (2022) 46(2):88–95. 10.1097/NPT.0000000000000383 35081081

[13] Huebner AC Lytle SR Doettl SM Plyler PN Thelin JT. Treatment of objective and subjective benign paroxysmal positional vertigo. J Am Acad Audiol. (2013) 24(7):600–6. 10.3766/jaaa.24.7.8 24047947

[14] McCaslin DL. Subjective BPPV: to reposition, or not to reposition, that is the question. J Am Acad Audiol. (2013) 24(7):534. 10.3766/jaaa.24.7.1. 24047940

[15] Moreno JLB Muñoz RC Matos YR Balboa IV Puértolas OC Ortega JA. Responses to the dix-hallpike test in primary care: a comparison between subjective and objective benign paroxysmal positional vertigo. Aten Primaria. (2021) 53(8):102023. 10.1016/j.aprim.2021.102023 34000460 PMC8141668

[16] Sivrice ME. Efficacy of epley maneuver on quality of life of elderly patients with subjective BPPV. J Int Adv Otol. (2020) 16(1):145–6. 10.5152/iao.2020.8135. 32401209 PMC7224438

[17] Uz U Uz D Akdal G Çelik O. Efficacy of epley maneuver on quality of life of elderly patients with subjective BPPV. J Int Adv Otol. (2019) 15(3):420–4. 10.5152/iao.2019.6483 31846923 PMC6937179

[18] Arrell R Manetta C Guthrie M Enam N. The prevalence of symptom reporting for benign paroxysmal positional vertigo in a traumatic brain injury population. Otol Neurotol. (2023) 44(2):172–6. 10.1097/MAO.0000000000003770 36624599

[19] Jumani K Powell J. Benign paroxysmal positional vertigo: management and its impact on falls. Ann Otol Rhinol Laryngol. (2017) 126(8):602–5. 10.1177/0003489417718847 28718303

[20] APTA Guide to Physical Therapist Practice 4.0. American Physical Therapy Association. Vol. 4. (2023).

[21] Kerber KA. Benign paroxysmal positional vertigo: opportunities squandered. Ann N Y Acad Sci. (2015) 1343:106–12. 10.1111/nyas.12721 25758295

[22] Jung JY Kim SH. Comparison between objective and subjective benign paroxysmal positional vertigo: clinical features and outcomes. Acta Otolaryngol. (2016) 136(12):1267–72. 10.1080/00016489.2016.1203990 27388229

[23] Herdman SJ. Advances in the treatment of vestibular disorders. Phys Ther. (1997) 77(6):602–18. 10.1093/ptj/77.6.602. 9184686

[24] Katsarkas A. Benign paroxysmal positional vertigo (BPPV): idiopathic versus post-traumatic. Acta Otolaryngol. (1999) 119(7):745–9. 10.1080/00016489950180360 10687929

[25] Agrawal Y Carey JP Della Santina CC Schubert MC Minor LB. Disorders of balance and vestibular function in US adults: data from the national health and nutrition examination survey, 2001-2004. Arch Intern Med. (2009) 169(10):938–44. 10.1001/archinternmed.2009.66 19468085

[26] Ballvé JL Carrillo-Muñoz R Rando-Matos Y Villar I Cunillera O Almeda J Effectiveness of the epley manoeuvre in posterior canal benign paroxysmal positional vertigo: a randomised clinical trial in primary care. Br J Gen Pract. (2019) 69(678):e52–60. 10.3399/bjgp18X700253 30510098 PMC6301349

[27] Chiarovano E Vidal PP Magnani C Lamas G Curthoys IS de Waele C. Absence of rotation perception during warm water caloric irrigation in some seniors with postural instability. Front Neurol. (2016) 7:4. 10.3389/fneur.2016.00004 26834699 PMC4725157

[28] Kovacs E Wang X Grill E. Economic burden of vertigo: a systematic review. Health Econ Rev. (2019) 9(1):37. 10.1186/s13561-019-0258-2 31883042 PMC6933936

[29] Enecke H Agus S Kuessner D Goodall G Strupp M. The burden and impact of vertigo: findings from the REVERT patient registry. Front Neurol. (2013) 4:136. 10.3389/fneur.2013.00136 24106487 PMC3788351

[30] U.S. Bureau of Labor Statistics. Occupational employment and wage statistics (2022). Available at: https://www.bls.gov/oes/current/oes291123.htm (Accessed April 18, 2023).

[31] Kerber KA Damschroder L McLaughlin T Brown DL Burke JF Telian SA Implementation of evidence-based practice for benign paroxysmal positional vertigo in the emergency department: a stepped-wedge randomized trial. Ann Emerg Med. (2020) 75(4):459–70. 10.1016/j.annemergmed.2019.09.017 31866170 PMC8161550

