# Supplementary material for: Surveying physical therapists' understanding of benign paroxysmal positional vertigo
Source: Front Rehabil Sci. 2023 Aug 17;4:1228453. doi: 10.3389/fresc.2023.1228453 (PMC10469676; doi:10.3389/fresc.2023.1228453)
Supplement: Supplementary file 1 [file Datasheet1.docx]

Appendix: Full survey

BPPV survey

Start of Block: Default Question Block

Q1 Welcome to the research study! You are being asked to participate since you have some experience with treating people with benign paroxysmal positional vertigo (BPPV). We are interested in your responses to 4 short clinical scenarios.
Please be assured that your responses will be kept completely confidential. The study should take you less than 5 minutes to complete. Your participation in this research is voluntary. You have the right to withdraw at any point during the study, for any reason, and without any prejudice. We will collect no identifiers other than zip code.
A breach of confidentiality is a minor risk, but we will do everything possible to reduce that risk. By clicking the button below, you acknowledge that your participation in the study is voluntary, you are 18 years of age, and that you are aware that you may choose to terminate your participation in the study at any time and for any reason. You can withdraw/terminate your participation by closing your browser and not completing the survey. We will use the data that has already been collected if you decide to close your browser. All responses to this survey are confidential.
If you would like more information about the survey, please contact the primary investigator: Susan L. Whitney, DPT, PhD, NCS, FAPTA at whitney@pitt.edu
Affiliation: University of Pittsburgh

- I consent to participate in this survey (1)
- I do not consent to participate in this survey (2)

End of Block: Default Question Block

Start of Block: Block 1

Q2 Do you diagnose/treat patients with benign paroxysmal positional vertigo (BPPV) in your practice?

- Yes (1)
- No (2)

Q3 If you suspect that the patient has BPPV, what do you do?

- Recommend Meclizine/Dramamine (1)
- Perform repositioning exercise as dictated by involved canal (2)
- Refer out to another provider (3)

Q14 Who would you refer the patient to? (check all that apply)

- Otolaryngology (1)
- Neurology (2)
- Primary Care Physician (3)
- Vestibular Physical Therapy (4)
- Audiology (5)
- Other (please specify) (6) __________________________________________________

End of Block: Block 1

Start of Block: Block 2

Q4 A 65-year-old male presents to your clinic with complaints of brief spinning dizzy spells from getting in and out of bed, looking up and down, walking, and physical activities in general. Would you assess this patient using positional testing (such as the Dix-Hallpike)?

- Never (1)
- Sometimes (2)
- About half the time (3)
- Most of the time (4)
- Always (5)

| Page Break |  |
| --- | --- |

Q5 When the patient returns the next visit following treatment for BPPV, he continues to complain of being off balance with slight non-spinning light-headedness in response to getting in and out of bed, looking up and down, walking, and physical activities in general. Would you reassess this patient using positional testing (such as the Dix-Hallpike)?

- Never (1)
- Sometimes (2)
- About half the time (3)
- Most of the time (4)
- Always (5)

| Page Break |  |
| --- | --- |

Q6 A patient reports spinning vertigo that lasts 10 seconds without nystagmus in the Dix-Hallpike position. What is your diagnosis?

- Functional dizziness (E.g. Persistent Postural Perceptual Dizziness, Mal de Debarquement Syndrome) (1)
- Vestibular migraine (2)
- Benign Paroxysmal Positional Vertigo (3)
- I am unable to make a PT diagnosis (4)

Q17 If you are unable to make a PT diagnosis, what would you do?

- Recommend Meclizine/Dramamine (4)
- Perform repositioning exercise as dictated by the involved canal (5)
- Refer out to another provider (6)

Q19 Who would you refer the patient to? (check all that apply)

- Otolaryngology (4)
- Neurology (5)
- Primary Care Physician (6)
- Vestibular Physical Therapy (7)
- Audiology (8)
- Other (please specify) (9)

| Page Break |  |
| --- | --- |

Q7 If you observe torsional upbeating nystagmus that fatigues in response to Dix-Hallpike head hanging, but they report no spinning. What is your diagnosis?

- Functional dizziness (E.g. Persistent Postural Perceptual Dizziness, Mal de Debarquement Syndrome) (1)
- Vestibular migraine (2)
- Benign Paroxysmal Positional Vertigo (3)
- I am unable to make a PT diagnosis (4)

Q20 If you are unable to make a PT diagnosis, what would you do?

- Recommend Meclizine/Dramamine (4)
- Perform repositioning exercise as dictated by the involved canal (5)
- Refer out to another provider (6)

Q21 Who would you refer the patient to? (check all that apply)

- Otolaryngology (4)
- Neurology (5)
- Primary Care Physician (6)
- Vestibular Physical Therapy (7)
- Audiology (8)
- Other (please specify) (9)

End of Block: Block 2

Start of Block: Block 3

Q9 Please check your current professional status

- Physical Therapist (1)
- Physical Therapist Assistant (2)
- Physical Therapy student (3)
- Physical Therapist Assistant student (4)

Q10 What is the primary focus of your practice?

- Academic/research (23)
- Acute care (24)
- Home Health (25)
- Inpatient rehabilitation (26)
- Oncology (27)
- Outpatient neurologic/vestibular (28)
- Outpatient orthopedics (29)
- Pediatrics (30)
- Skilled nursing facility (31)
- Sports (32)
- Women's Health (33)
- In school/I don't have a focus of practice yet (34)

Q15 How many years have you practiced as a PT or PTA?

________________________________________________________________

Q12 Do you treat more than 20 different patients with BPPV per year?

- No (1)
- Yes (2)

Q16 What zip code do you primarily practice in?

________________________________________________________________

End of Block: Block 3
